# Systematic review protocol for evaluating the ecological effects of ground-mounted photovoltaic facilities on faunistic biodiversity in grasslands^✰^

**DOI:** 10.1016/j.mex.2026.104209

**Published:** 2026-10-14

**Authors:** Wiltrut Koppensteiner, Arndt Piayda, Hanna Rae Martens, Felix Zitzmann, Kirsten Brandt, Franziska Tanneberger, Bärbel Tiemeyer

**Affiliations:** a Thünen Institute of Climate-Smart Agriculture, Bundesallee 65, Braunschweig, 38116, Germany; b University of Greifswald, Partner in the Greifswald Mire Centre, Soldmannstraße 15, Greifswald, 17489, Germany; c Institute of Environmental Planning, Leibniz University Hannover, Herrenhäuser Straße 2, Hannover, 30419, Germany

**Keywords:** Peatland, Solar power, Renewable energy, Rewetted peatland PV, PECO, Benchmark list, Systematic search string, CADIMA, Birds, Bats, Pollinators, Semi-aquatic insects, Monitoring

## Abstract

Biodiversity loss and climate change are two interconnected crises, but are often addressed separately in policy and land-use planning, which creates trade-offs, especially regarding renewable energy systems. As photovoltaic (PV) installations expand globally, concerns about how this land-use change affects biodiversity are growing. Few studies have examined the effects of ground-mounted PV facilities on biodiversity, and evidence remains limited, especially for faunistic biodiversity. Thus, we defined this systematic review protocol in January 2024, according to PRISMA guidelines and applying the PECO framework to guide a relatedsystematic review. Especially, development of adequate search string and validation of its comprehensiveness crucial: A systematic search string was developed using the R packages “litsearchr” (Grames et al. 2019) with an additional pruning exercise which improved the search string result. And, a workflow was developed to validate the comprehensiveness of different search strings using a benchmark list of publications and publications found by applying a search string from a published systematic map with a broader research question. In addition, the application and screening using the software CADIMA increased transparency and reproducibility of the method. Therefore, this protocol could serve as a blueprint for similar research questions involving other ecosystems by adapting published methods to a specific ecological question.

## Graphical abstract

**Figure:**
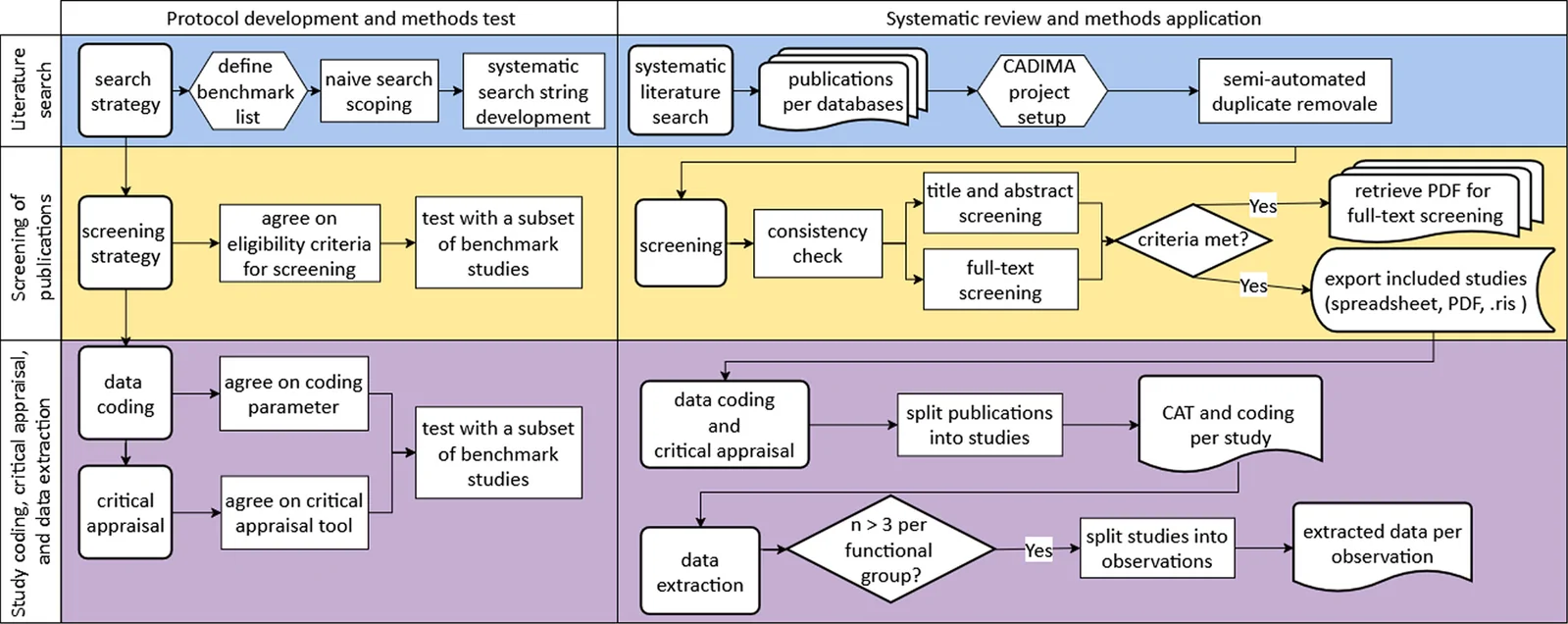

## Specifications table

**Table:** 

**Subject area**	Environmental Science
**More specific subject area**	Ecology and Conservation
**Name of your protocol**	Systematic review protocol for evaluating the ecological effects of ground-mounted photovoltaic facilities on faunistic biodiversity in grasslands
**Reagents/tools**	none
**Experimental design**	Systematic Review Protocol
**Trial registration**	none
**Ethics**	none
**Value of the Protocol**	• First systematic review protocol for the effect of ground-mounted PV systems on grasslands. • Photovoltaic installations expand worldwide, which raises concerns on the effects on biodiversity. • The protocol adheres to PRISMA guidelines and uses the PECO framework as well as predefined eligibility criteria, thus allowing a transparent synthesis of available evidence. • The protocol could serve as a blueprint for similar research questions involving other ecosystems by adapting published methods to a specific ecological question: ✓ development of coding and data extraction spreadsheets to improve the reproducibility of the analysis for the systematic review ✓ application of the R packages *litsearchr* and recommendations by Grames et al. [1] for developing the systematic search string, and extending the method by pruning exercises which improved search string result ✓ development and application of a validation workflow to test for comprehensiveness of different search strings using a benchmark list of publications and publications found by applying a published search string for a broader research question [2]

## Background

Climate change and biodiversity loss are among the most severe global crises today. Both are predominantly driven by anthropogenic activities, and their interactions are widely acknowledged [3,4]. Nevertheless, climate change mitigation and biodiversity conservation have often been addressed separately in policy and land-use planning, resulting in a potential trade-off, the so-called “green-green dilemma” in the context ​of renewable energy expansion [5]. One of the most promising renewable energy types are photovoltaic (PV) systems. Especially, improving the compatibility of ground-mounted PV systems with biodiversity conservation is increasingly discussed [[6], [7], [8]].

One new ground-mounted PV system is “Rewetted Peatland PV” which promises to combines the reduction of greenhouse gas (GHG) emissions from drained organic soils with biodiversity enhancement. In Germany, the amendment of the Renewable Energy Act in 2023 allows the installation of PV systems on rewetted organic soils previously drained for agriculture. Wet peatlands are most often open landscapes of high importance for biodiversity conservation, as they provide habitats for many threatened and highly specialized species. Consequently, the expansion of Rewetted Peatland PV systems raises concerns regarding their potential impacts on peatland-specific biodiversity [9].

A growing number of studies have investigated biodiversity responses to ground-mounted PV systems on mineral soils [2,6,8]. In 2023, a first systematic map [2] was published in accordance with the guidelines of a systematic literature search and screening (e.g., [10,11]). Lafitte et al. [2] focused on evidence regarding the effects of wild terrestrial and semi-aquatic fauna and included peer- and French non-peer-reviewed publications. The study provides important insights but is insufficient to derive recommendations for mitigation measures for vertebrate and invertebrate species, either for PV installations on grassland or for Rewetted Peatland PV in Germany. Consequently, existing recommendations (e.g., [12]) are still based on non-systematic reviews, often including PV systems that are not used in Germany or Europe. From a conservation perspective, there is an urgent need to systematically synthesize the available evidence on the effects of ground-mounted PV installations on faunistic biodiversity and to critically assess their implications for emerging new PV systems on peatlands. Addressing this knowledge gap is essential to inform policy and integrated planning approaches that seek to resolve the “green–green dilemma” and align ground-mounted PV expansion with biodiversity conservation goals.

Therefore, the main objective of this protocol is to enable a systematic review on the effects of ground-mounted PV installations on faunistic biodiversity in grasslands and discuss the transferability of these results to Rewetted Peatland PV. Our methods follow the systematic review guidelines by using a PECO framework and a systematic search and screening strategy (e.g., [11,13,14]). The primary question guiding this review protocol was: What are the effects of Grassland PV on terrestrial and semi-aquatic faunistic biodiversity?

## Description of protocol

Our protocol follows the systematic review guidelines by using a PECO framework, the guidelines for Preferred Reporting Items for Systematic Reviews and Meta-Analyses (PRISMA, [11,15]) and a systematic search and screening strategy (e.g., [10,11,13,16]). Most of the recommended methodological steps of full systematic reviews (e.g., [14,17,18]) were applied. Limitations are discussed in the corresponding section.

− *a priori* eligibility criteria (inclusion/ exclusion) based on PECO framework were defined
− *a priori* defined criteria and methods for screening, coding, data extraction, critical appraisal and synthesis were used
− The review team agreed on this review protocol defining all steps before conducting the whole review, although it was not published beforehand
− A recommended approach to create a systematic search string [1] was used, but was adapted to fit our demands
− A search for peer-reviewed and grey literature was performed in diverse databases and specific websites
− Comprehensiveness was tested using predefined benchmark list of publications and publications found when applying a published search string for a broader research question than ours [2]
− All steps were tested and consistency checks were performed using a trial dataset for all screening stages, data extraction, and critical appraisal.

a) **Workflow**

Before agreeing on the protocol of the systematic review, the lead reviewer successfully completed an online course on the methodology of systematic reviews from CEE (https://systematicreviewmethods.github.io/index.html). Based on the recommendation, the team roles of chair reviewer (supervisor), lead reviewer, and reviewer were assigned, and the PRISMA workflow was established (Fig. 1). The systematic review was performed in three phases with discussion and adaptation loops: testing, training, and applying [13].

**Fig. 1:**
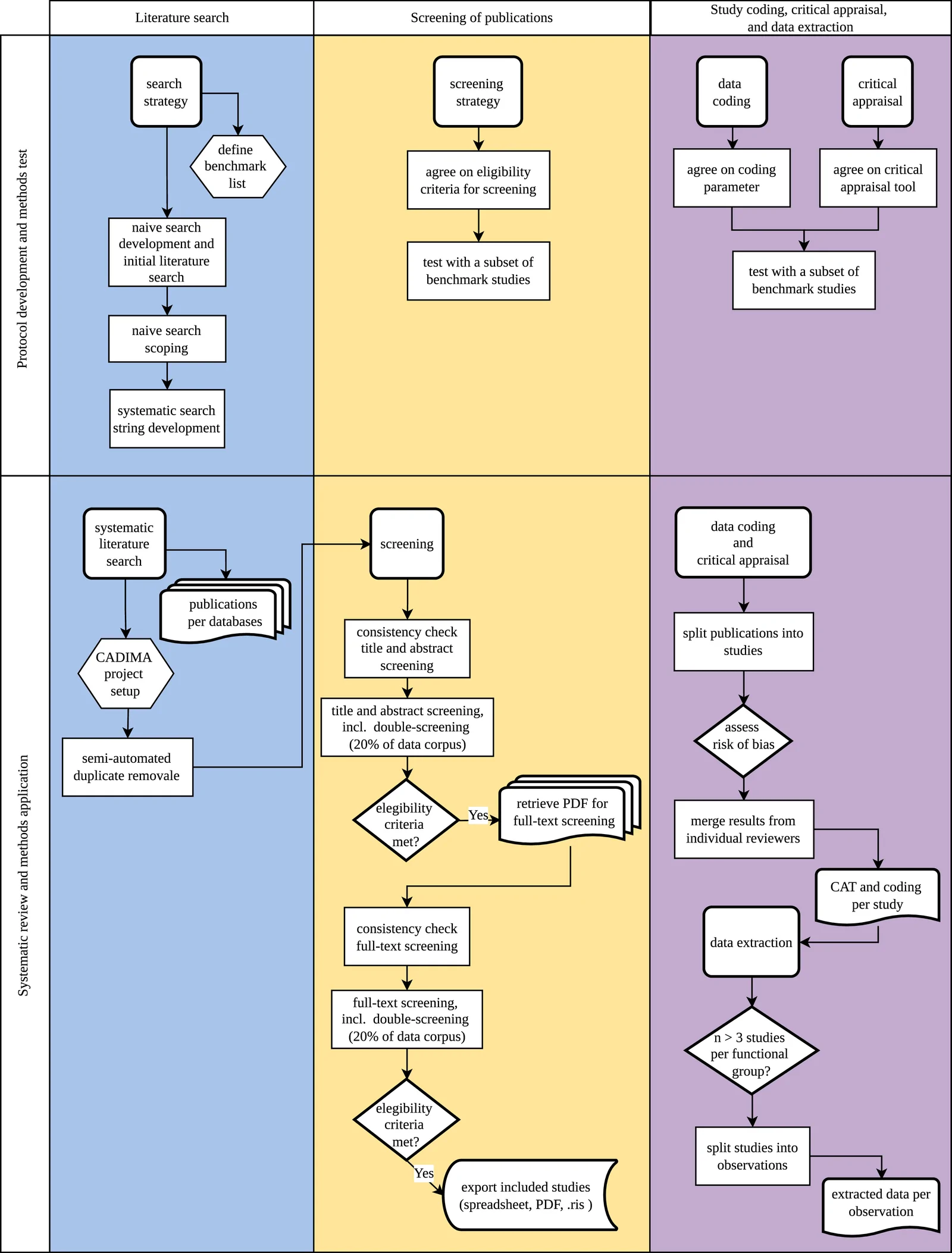
Flow diagram of protocol development and the application of protocol in the systematic review.

b) **Eligibility criteria**

The primary research question was translated into the PECO framework (population – exposure– comparator– outcome; [19,20]):

− Population: all terrestrial and semi-aquatic vertebrate and invertebrate species (e.g., birds, bats, insects, arachnids, other arthropods), excluding plant, fungal, microbial and aquatic species, as well as humans and domestic or cultivated species (e.g., sheep, honey bees)
− Exposure: Installation of all scales of ground-mounted PV systems using designs applicable in grassland habitats, excluding PV systems on buildings, in desert habitats, and concentrated solar power (CSP)
− Comparator: Studies with references of any kind, like population exposed to PV facilities or PV panels with one unexposed, population before and after exposure, studies comparing different types of PV panels or PV facilities
− Outcome: All outcome related to the studied target population (e.g., behavior, habitat use or population parameters), excluding biotic and abiotic parameters that are only related to the studied habitat or ecosystem (e.g., precipitation, temperature)

Further, eligibility criteria were defined for the screening of publications based on the PECO framework, following recommendations and guidelines for systematic reviews [10,14,21]. For title and abstract screening of publications, the eligibility criteria of “population” and “exposure” were mandatory, whereas the criteria of “outcome” and “comparator” were optional (Table 1). In addition, criteria for language and document content were included for full-text screening. Only English and German publications were included at the full-text stage because of the linguistic abilities of the team members. Furthermore, empirical studies or observations, reviews, concepts, meta-analyses, and modelling publications were included when they provided extractable data for faunistic biodiversity. All geographical locations were included, and the restriction to grassland habitats was made during full-text screening because habitat information is unlikely to be defined in the title or abstract of publications. The “comparator” criterion included all types of comparisons, such as populations exposed to PV facilities or PV panels compared with one unexposed population, populations before and after exposure, and studies comparing different types of PV panels or PV facilities. All reported outcomes were included as long as they focused on terrestrial or semi-aquatic fauna connected to ground-mounted PV facilities or PV panels after construction.

**Table 1:** List of eligibility criteria based on the PECO framework and additional criteria, with information on relevance at the defined screening stages, “title and abstract” and “full-text.”.

**criteria**	**screening stage**	**include**	**exclude**
**population**	all stages	all terrestrial and semi- aquatic vertebrate and invertebrate species	plant species, aquatic species, humans and domestic or cultivated species
**exposure**	all stages	ground-mounted PV facilities, and PV panels (incl. agrivoltaics, and simulated PV panels) after construction independent of its scale with connection to grassland habitats	solar-thermal panels, PV panels on buildings, Concentrated Solar Parks (CSP), glacial PV installations, floating PV installations, studies on the life cycle of PV panels or solar cells, studies focusing on construction, repowering or dismantlement of PV panels
**outcome**	title and abstract (optional), full text (obligated)	primary studies showing all outcomes related to the studied population (behaviour, habitat use or population), including reviews and theoretical concepts which synthesis the above outcomes with reported primary data	abiotic parameters that are only related to the studied habitats or ecosystems without direct connection to the population or economical, technical, or policy assessments, publications without reported primary data
**comparator**	title and abstract (optional), full-text (obligated)	studies with a type of comparison	studies without any type of comparison
**language**	full-text (obligated)	English, German	all other languages
**document content**	full-text (obligated)	empirical, observational or modelling studies, meta-analyses, reviews, concepts including peer-reviewed and grey literature, and preprints	policy documents, law or opinion publications and concepts with no primary data, posters, conference papers and abstracts

c) **Search strategy**

### Comprehensiveness of search

Prior to search string development, a benchmark list of relevant publications based on the experiences of review team members and additional recommendations was defined. Using this benchmark list, the comprehensiveness of our literature search was validated. In addition, our search results were compared with the provided list of included publications from Lafitte et al. [2].

The pre-defined benchmark list included 28 publications (Supplementary Material S1). The publications were mostly in English, except for one publication in German. Most publications were found in the Web of Science Core Collection (WOSCC; access of the Thünen Institute), but three publications were found only in the Scopus database.

### Development of naïve search string

Our search string was developed using the PECO framework and the R package *litsearchr* [1]. First, a naïve search string was built based on the primary question. Therefore, possible search terms for population and exposure were identified by the review team, as well as terms that may need to be excluded (Supplementary Material S2). Second, using the first naïve search string, a scoping exercise was performed on January 15 in 2024, with WOSCC and results were discussed within the review team to optimize the naïve search. The scoping results are provided in the supplements (Supplementary Material S2). Subsequently, the six most promising search strings from this scoping result were compared with publications from the benchmark list and publications identified using the search string from Lafitte et al. [2] in WOSCC on the same day (n = 3670 publications). The finding rate and inverse finding rate were calculated: The finding rate refers to the proportion of publications identified from either the benchmark list or through the application of the search string by Lafitte et al. [2] using the naïve search string. The inverse finding rate is defined as the percentage of similar publications with the search string from Lafitte et al. [2] out of the total publications found using the naïve search string (Table 2).

**Table 2:** Comparison of naïve search string scoping with the publications found using the search string from Lafitte et al. [2] and the benchmark list of publications (Supplementary Material S1). Example calculation of inverse finding rate = ((762/772)*100), finding rate = ((762/3670)*100). Text in bold = additional search terms.

search string	search string results (comments)	number of similar publications of naïve search with search string applications from Lafitte et al. [2] (inverse finding rate and finding rate [%])	number of benchmark list publications in naïve search (finding rate [%])
TS = (((photovoltaic$ OR “solar power” OR “solar power plant$” OR “solar park$” OR “solar farm$” OR “solar facilit*” OR “solar panel”) NOT (roof* OR house$ OR building$ OR “solar cell$” OR “concentrated solar” OR “concentrating solar”)) AND (biodivers* OR wildlife OR fauna OR mammal$ OR bird$ OR avian OR chiroptera$ OR reptile$ OR amphibian$ OR insect$ OR arachnid$ OR arthropod$ OR invertebrate$ OR **“conservation”**))	**565 WOSCC hits** (exclude ecological, include conservation; with NOT)	254 (45%* and 7%)	17 (61%)
TS = ((photovoltaic$ OR “solar power” OR “solar power plant$” OR “solar park$” OR “solar farm$” OR “solar facilit*” OR “solar panel”) AND (biodivers* OR wildlife OR fauna OR mammal$ OR bird$ OR avian OR chiroptera$ OR reptile$ OR amphibian$ OR insect$ OR arachnid$ OR arthropod$ OR invertebrate$ OR **“conservation”**))	**938 WOSCC hits** (exclude ecological, include conservation; without NOT)	359 (38%* and 10%)	19 (68%)
TS = (((photovoltaic$ OR “solar power” OR “solar power plant$” OR “solar park$” OR “solar farm$” OR “solar facilit*” OR “solar panel”) NOT (roof* OR house$ OR building$ OR “solar cell$” OR “concentrated solar” OR “concentrating solar”)) AND (biodivers* OR wildlife OR fauna OR mammal$ OR bird$ OR avian OR chiroptera$ OR reptile$ OR amphibian$ OR insect$ OR arachnid$ OR arthropod$ OR invertebrate$ OR **“ecological”**))	**553 WOSCC** (exclude conservation, include ecological; with NOT)	545 (99%* and 15%)	17 (61%)
TS = ((photovoltaic$ OR “solar power” OR “solar power plant$” OR “solar park$” OR “solar farm$” OR “solar facilit*” OR “solar panel”) AND (biodivers* OR wildlife OR fauna OR mammal$ OR bird$ OR avian OR chiroptera$ OR reptile$ OR amphibian$ OR insect$ OR arachnid$ OR arthropod$ OR invertebrate$ OR **“ecological”**))	**772 WOSCC** (exclude conservation, include ecological; without NOT)	762 (99%* and 21%)	19 (68%)
TS = (((photovoltaic$ OR “solar power” OR “solar power plant$” OR “solar park$” OR “solar farm$” OR “solar facilit*” OR “solar panel”) NOT (roof* OR house$ OR building$ OR “solar cell$” OR “concentrated solar” OR “concentrating solar”)) AND (biodivers* OR wildlife OR fauna OR mammal$ OR bird$ OR avian OR chiroptera$ OR reptile$ OR amphibian$ OR insect$ OR arachnid$ OR arthropod$ OR invertebrate$))	**219 WOSCC** (exclude ecological and conservation; with NOT)	214 (98%* and 6%)	17 (61%)
TS = ((photovoltaic$ OR “solar power” OR “solar power plant$” OR “solar park$” OR “solar farm$” OR “solar facilit*” OR “solar panel") AND (biodivers* OR wildlife OR fauna OR mammal$ OR bird$ OR avian OR chiroptera$ OR reptile$ OR amphibian$ OR insect$ OR arachnid$ OR arthropod$ OR invertebrate$))	**307 WOSCC** (exclude ecological and conservation; without NOT)	301 (98%* and 8%)	19 (68%)

The application of the naïve search strings resulted in a 61% finding rate of benchmark publications when the NOT clause was applied and 68% without the NOT clause (Table 2). The use of the defined NOT clauses excluded Barré et al. [22] and Lafitte et al. [2] and was therefore not pursued further. It is reasonable that a 100% finding rate of benchmark list publications could not be obtained with a search in WOSCC only, as not all relevant publications are included here [[23], [24], [25]]. The comparison with the application of the search string from Lafitte et al. [2] resulted in 38% – 99% similar publications of our naïve search (Table 2). Nevertheless, many irrelevant papers for our review could be excluded compared to the search string application from Lafitte et al. [2], given the narrower primary research question than that of Lafitte et al. [2]. Thus, the comparison with the benchmark list as well as the results using the Lafitte et al. [2] search string provided a guideline for which search string to choose, in addition to our scoping exercise. The scoping and comprehensiveness checks were discussed within the review team. Therefore, the most promising search string with the best balance between sensitivity and specificity for our search question was the search string, which resulted in 772 hits in WOSCC:

TS = ((photovoltaic$ OR “solar power” OR “solar power plant$” OR “solar park$” OR “solar farm$” OR “solar facilit*” OR “solar panel”) AND (biodivers* OR wildlife OR fauna OR mammal$ OR bird$ OR avian OR chiroptera$ OR reptile$ OR amphibian$ OR insect$ OR arachnid$ OR arthropod$ OR invertebrate$ OR “ecological”))

Next, the naïve search was performed in the databases Biological Abstract (BA), CAB Abstract (CA), and WOSCC (hereafter referred to as naïve search results). A total of 1428 publications were found (after de-duplication by exact title): BA with 546, CAB Abstract with 421, and WOSCC with 772 publications.

## Development of systematic search string

The systematic search string was developed using the naïve search results by identifying relevant keywords using the R package *litsearchr* [1]. However, after the first trial, an additional step of pruning was included in the systematic search string development: The titles of the naïve search results were screened for irrelevant and misleading publications that might bias the application of the keyword search using *litsearchr* (e.g., animal tracking system, bird droppings on panels, life cycle assessments, economic publications and (GHG) emissions, and photovoltaic effect in hornets). The assessment was based on the Population – Exposure and titles were included in cases of uncertainty, as recommended by the CEE guidelines [10]. Pruning was performed by two reviewers in 45–30-min intervals for efficiency, and decisions were discussed and resolved immediately in cases of conflict. After pruning, the dataset for the *litsearchr* analysis consisted of 224 publications (inclusion rate: 15%; Supplementary Material S1). This corresponds with the criteria for a narrow search by Grames et al. [1].

Based on the pruned naïve search results, potential keywords were identified using *litsearchr* for keywords chosen by the authors (method = "tagged", min_freq = 2, min_n = 1). Only eight publications did not provide keywords by the authors. In addition, potential keywords were added from titles using the Rapid Automatic Keyword Extraction (RAKE) provided in *litsearchr* (method= "fakerake", min_freq = 3, min_n = 2) and applied the package-specific “stopwords tool = English”. Keywords from the abstract were not included because initial trials showed no improvement but resulted in more misleading keywords. Subsequently, a document feature matrix was built (min_studies = 2, min_occ = 2), which showed that most search terms are located in one cluster, although some terms are located at the edge of the network (Supplementary Material S2). These terms might not be as important as the terms in the center; however, all terms needed to be evaluated manually by the authors [1].

Next, the rank node strength method was applied to find an adequate cutoff point, as described by Grames et al. [1]. Here, a threshold of 70% of the total strength of the network was used and the cumulative instead of the changing-point method was applied to the cut-off function (Supplementary Material S2). However, based on a comparison between all extracted keywords and the reduced keywords using the cut-off method, we decided against the latter because relevant keywords were excluded. All extracted keywords were grouped into the concept groups „Population” and “Exposure” and added some important search terms manually (Supplementary Material S2). Subsequently, the systematic search string was written and stemming was applied using an asterisk for at least four characters in *litsearchr*. Unfortunately, the systematically built search string resulted in 28,000 publications when applied in WOSCC, with many misleading or irrelevant publications. Therefore, the search string was adapted based on our experiences from the naïve search scoping exercise, especially the stemmed terms needing critical revision. The complete adaptation of the systematic search string is provided in Supplementary Material S2, and Table 3 highlights the most important steps.

**Table 3:** Adaptation steps of the systematic built search string from litsearchr [1].

**adaptation step**	**search string**	**hits in WOSCC**
**Naïve search string (scoping result)**	TS = ((photovoltaic$ OR “solar power” OR “solar power plant$” OR “solar park$” OR “solar farm$” OR “solar facilit*” OR “solar panel”) AND (biodivers* OR wildlife OR fauna OR mammal$ OR bird$ OR avian OR chiroptera$ OR reptile$ OR amphibian$ OR insect$ OR arachnid$ OR arthropod$ OR invertebrate$ OR “ecological”))	WOSCC: 772
**Original *litsearchr* search string**	TS = (("solar* facil*" OR "solar* park*" OR "solar* power* park*" OR "solar* plant*" OR "solar* power* plant*" OR "agrivolta* system*" OR agrivolta* OR "float* solar*" OR photovolta* OR "solar* energi* facil*" OR "solar* farm*" OR "solar* panel*" OR "util* scale* solar*" OR "utility-scal* solar*") AND (fauna* OR bird* OR avian* OR amphibian* OR chiroptera* OR reptil* OR insect* OR "aquat* anim*" OR "aquat* organ*" OR biodivers* OR conserv* OR ecolog* OR ecosystem* OR environ* OR speci* OR "wild* anim*" OR wildlif*))	WOSCC: 28,173
**Adapted *litsearchr* search string**	TS = (("solar facil*" OR "solar park*" OR "solar power park*" OR "solar plant*" OR "solar power plant*" OR "agrivolta* system*" OR agrivolta* OR photovolta* OR "solar energy facil*" OR "solar farm*" OR "solar panel*" OR "utility scale* solar" OR "utility-scale* solar") AND (fauna OR bird* OR avian OR amphibian* OR chiroptera OR reptil* OR insect* OR "aquat* anim*" OR biodivers* OR ecolog* OR "wild anim*" OR wildlife) NOT “life cycle”)	WOSCC: 800
**Adapted litsearchr search string with additional term**	TS = (biodivers* AND ("solar* facil*" OR "solar* park*" OR "solar* power* park*" OR "solar* plant*" OR "solar* power* plant*" OR "agrivolta* system*" OR agrivolta* OR "float* solar*" OR photovolta* OR "solar* energi* facil*" OR "solar* farm*" OR "solar* panel*" OR "util* scale* solar*" OR "utility-scal* solar*") AND (fauna* OR bird* OR avian* OR amphibian* OR chiroptera* OR reptil* OR insect* OR "aquat* anim*" OR "aquat* organ*" OR biodivers* OR conserv* OR ecolog* OR ecosystem* OR environ* OR speci* OR "wild* anim*" OR wildlif*))	WOSCC: 108

Again, comprehensiveness checks were conducted for i) the different search strings applied in WOSCC with the results from Lafitte et al. [2], ii) the pre-defined benchmark list, iii) and the titles from our pruned naïve search (Table 4). The comparison indicates that our naïve search string was already better than the systematic search strings. The most promising systematic search string was the adapted *litsearchr* search string, resulting in 800 publications in WOSCC (Table 3): TS = (("solar facil*" OR "solar park*" OR "solar power park*" OR "solar plant*" OR "solar power plant*" OR "agrivolta* system*" OR agrivolta* OR photovolta* OR "solar energy facil*" OR "solar farm*" OR "solar panel*" OR "utility scale* solar" OR "utility-scale* solar") AND (fauna OR bird* OR avian OR amphibian* OR chiroptera OR reptil* OR insect* OR "aquat* anim*" OR biodivers* OR ecolog* OR "wild anim*" OR wildlife) NOT “life cycle”)

**Table 4:** Comparison of search string adaptation steps with the publications found using the search string from Lafitte et al. [2], the benchmark list of publications, and the pruned publications from the naïve search string development (Supplementary Material S2). The inverse finding rate and finding rate are shown in Table 2.

**adaptation step** (see Table 3)	**number of similar publications of search steps with search string applications from Lafitte et al.** [2] **(inverse finding rate [%])**	**number benchmark list publications in search steps (finding rate [%])**	**number pruned publications in search steps (finding rate [%])**
naïve search string (scoping decision)	762 (**99%**)	19 (68%)	772 (100**%**)
original *litsearchr* search string ***	951 (19%)	25 (89%)	509 (66%)
adapted *litsearchr* search string	768 (**86%**)	22 (**79%**)	628 (**81%**)
adapted *litsearchr* search string with additional term	102 (94%)	11 (39%)	92 (12%)
naïve search string with additional term	103 (96%)	10 (36%)	105 (14%)

The adapted *litsearchr* search string was applied in WOSCC, BA, and CAB and compared the results with the pruning decision of the naïve search. A total of 1403 publications were retrieved with the systematic search string (after de-duplication by exact title): BA with 548, CAB Abstract with 373, and WOSCC with 800 hits. The application of the adapted systematic search string (hereafter, “search string”) likely minimize potential bias in search string development and was therefore used in the connected systematic review (GECCO-D-26-01145).

The adapted systematic search string was also validated by comparing it with the publications found using the naïve search results. Most publications were identified using both search strings; however, 582 additional publications were identified using the systematic search string (Fig. 2). The additional publications were screened based on titles using the same methods as those used to prune the naïve search string. The searches using the pruned naïve search string and the adapted systematic search string yielded similar results for the number of relevant (naïve vs. systematic search: 116 vs. 121), uncertain (108 vs. 107), and irrelevant (1204 vs. 1175) publications based on title screening.

**Fig. 2:**
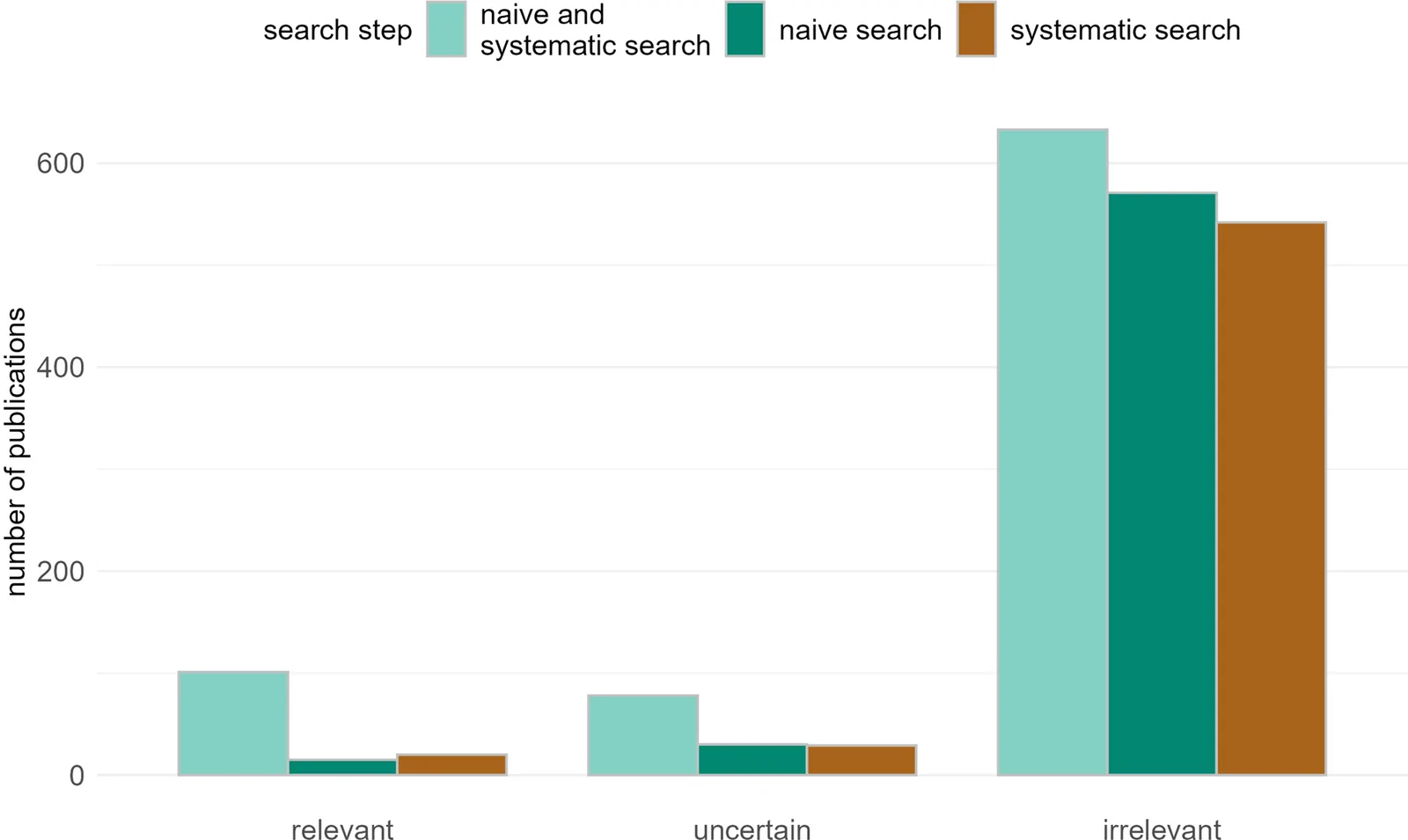
Comparison of the rapid screening of naïve search string and systematic search string results in the Web of Science Core Collection, Biological Abstract, and CAB Abstract databases.

d) **Information source**

A comprehensive literature search was performed on March 13, 2024, using the adapted systematic search string in the following multiple databases for peer-reviewed publications to minimize selection bias” [10,13,14]: Biological Abstract (BA), BIOSI Citation Index (BCI), CAB Abstract (CAB), Web of Science Core Collection (WOSCC), and Scopus (SCP). In addition, non-peer-reviewed (“grey literature”) was searched in the following websites and web-libraries in English and German: ProQuest, Google Scholar, BfN e-repositorium (https://bfn.bsz-bw.de/home), publications from the Greifswald Mire Center (GMC; https://greifswaldmoor.de/publications.html), and Kompentenzzentrum Naturschutz und Energiewende” (KNE; https://www.naturschutz-energiewende.de/fachwissen/publikationen/). Publications of all years and geographic locations were included. A list of database-specific search strings with the number of found publications and the date of the search is provided in Supplementary Material S2.

e) **Study selection process**

All publications were screened by title and abstract, and full-text based on the a priori defined criteria and the agreed workflow (Table 1, Fig. 1).

First, the general workflow for title and abstract screening and full-text screening was tested and discussed using spreadsheets and eight publications from the benchmark list (Fig. 1). For the screening of the whole data corpus, the software CADIMA ([27]; https://www.cadima.info/, accessed November 13, 2025) was used. The reasons for this choice were the practicability, transparency, and documentation of the software, as well as its efficiency during the screening process. All authors have access to the CADIMA project (“Evidences regarding the effects of ground-mounted solar parks”).

Therefore, the literature search results were uploaded, and the predefined eligibility criteria (Table 1) were added in a created CADIMA project, and the semi-automated deduplication of CADIMA was applied. In cases of uncertainty at title and abstract screening, the publications were marked as “uncertain” and included in full-text screening, as recommended by the CEE guidelines [10]. The screening team consisted of five reviewers (AP, HM FZ, KB, WK). Methodical issues were discussed and resolved by all authors.

Next, consistency checks for title and abstract screening (n = 150 publications) and consecutively full-text screening (n = 15 publications) were performed by the entire screening team. A Fleiss’ kappa coefficient value of 50% was defined beforehand as acceptable because of the number of reviewers [10]. This ensured a reproducible and reliable screening process [21]. For the complete study selection process, 20% of the publications will be screened by two reviewers (double-screening approach). Inconsistencies were discussed and resolved within the reviewer team. It was ensured that no reviewer screened their own publications. The screening steps and results are illustrated in a flow diagram based on PRISMA in the accompanying systematic review (see co-submission of systematic review to Global Ecology and Conservation). Linked articles were documented using the unique publication identifier created by CADIMA [10]. The results from the screening process were exported from CADIMA on March 31, 2025.

f) **Coding and data extraction**

Similar to the screening process, the coding of included studies was tested beforehand by all authors and discussed using spreadsheets and eight publications from the benchmark list. The coding process and categories were discussed with the entire team, and the template was adapted.

After full-text screening, the included publications were assigned to one review team member based on their expertise and field of research. In the template, four publications were already coded as examples to simplify the coding process for the review team.

At the coding stage, the publications were classified into individual studies based on different studied taxa and/or locations and/or PV types. The categories and descriptions are provided in Table 5, and the coding results are provided in the systematic review (see co-submission of systematic review to Global Ecology and Conservation).

**Table 5:** presents the study coding data categories and descriptions for the publications included after full-text screening.

**data category**	**descriptions**
bibliographic information	article ID, study ID, observation ID, article ID of linked publication (optional), author, title, year, language, document type, document content
review information	acronym of reviewer, summary of topic in reviewer’s own words
description of population	name of studied taxon as cited by authors, level of studied taxon (species to order), assessment of taxon’s relevance for the systematic review
description of exposure	installed vs. simulated solar facilities, construction year, number of studied solar facility sites, size [ha] of studied solar facilities, scale of PV installation [MWh], tracked vs. fixed solar panels installed, distance between PV panel rows [unit in paper], area used for PV panels [unit in paper], height of panel as described in paper (e.g. min. or max. panel height), width of panel as described in paper, fenced vs. unfenced solar facility, type of management, information on the surrounding habitat or ecosystem, information of additional actions for biodiversity
description of comparator	comparator as described by authors, assessment of comparator’s relevance for the systematic review
description of outcome	outcome as described by authors, assessment of outcome’s relevance for the systematic review
description of study design	number of study sites, total area of study site [unit in paper], year(s) of the study or survey, habitat of study area before solar facility installation, habitat of study area after solar facility installation, habitat of reference site, method used for taxon study (invasive vs. passive sampling methods), number of survey times or dates, duration of study and the sampling, sampling intervals, type of study (experiment vs. observation), location of study and/or study sites
additional information	information if data for data extraction is available within the manuscript or the supplements, information of the data location for data extraction (e.g., no. page, no. table), place for comments if needed (optional), web link of the supplement of the publication (optional), storage path of the supplement on reviewer’s PC and/or Cloud

To conduct a more detailed analysis of the effects of solar facilities and/or PV panels on the studied taxa and to gain insights into the potential driving factors of the effect direction (positive, neutral, mixed, or negative), additional data from the results sections and online supplements of the publications were extracted using spreadsheets and online tools (e.g., PDF24 https://www.pdf24.org/de/, WebPlotDigitizer V4, https://apps.automeris.io/wpd4/, Engauge Digitizer 4.1 https://engauge-digitizer.software.informer.com/4.1/). Data extraction was conducted by one reviewer for data homogenization purposes.

Data extraction was performed using predefined spreadsheets in Excel. Here, the included studies were split into unique observations based on the reported effects of species or species groups and habitats. Additional data was extracted on the response variable (e.g., species richness, abundance, or specific species), reported effect and sample sizes, performed statistical tests and the level of significance, the authors' conclusion, their reported overall effect on the studied taxa, as well as mentioned mitigation measures and knowledge gaps. Based on this information, the observations were assigned to an aggregated effect direction (positive, neutral, negative, mixed), if possible. The extraction columns are provided in Table 6, and the data extraction results are provided in the related systematic review.

**Table 6:** Presents the data extraction categories and descriptions for publications included after coding.

**Column**	**Description**
id.observation	unique observation ID: split by species, if results for species is given in manuscript
luc.type	land-use change type (e.g., grassland to PV site)
studied.species	studied species (separate column per species, if results for species is given in manuscript)
response.variable	reported ecological response of studied species (e. g, abundance, diversity, etc.)
explanatory.variable	reported explanatory variable for ecological response of studied species
effect.size	extracted effect size (e.g., LRR, Hedge's g, percentage, median, SD, SE, etc.)
sample.size	sample size per species
statistics	description of statistical analysis
effect.direction	manual categories: negative, neutral, positive, mixed
effect.sign	TRUE/ FALSE (effect is reported significance)
effect.interpretation.source	source of data extraction/interpretation of effect: textual, effect_size, figure_only/ mixed_results
effect.source.location	location of data extraction (e.g., Fig. 1, results paragraph 1)
effect.direction.notes	text about effect direction interpretation (used for manuscript later)
authors.conclusion	textual extraction of authors' conclusion
overall.effect.reported	textual extraction of authors' overall effects
disc.overall.effect.authors	textual extraction of authors' reasoning for overall effects
mitigation.recom	text about reported mitigation measures to promote PV sites for (faunistic) biodiversity
know.gaps	text about reported knowledge gaps regarding the effects of PV sites on (faunistic) biodiversity
comments	additional comments
critics.reviewer	critics on derived conclusions by authors for this review

g) **Critical appraisal**

The Critical Appraisal Tool (CAT) developed by Lafitte et al. [26] was used to assess the specific and overall risks of bias of the included studies. The decision to apply this tool instead of developing our own was based on the fit to our research question and the initial development of the CAT for a similar, although broader, data corpus. The CAT distinguished between eight specific risks of bias, which were then summarized within an overall risk of bias. Three levels of risk of bias were defined: “low”, “high”, and “unclear” (Fig. 3). The assessment of the specific risks of bias was performed by two review members answering the eight questions defined by Lafitte et al. [26]. In case of inconsistencies, the two reviewer members discussed their answers per study and agreed on an assessment. The overall risk of bias was then defined as follows:

− all specific risks of bias are low, then the overall risk of bias is low
− once one specific risk of bias is high, then the overall risk of bias is high
− if most specific risks of bias are low, but one is unclear, then the overall risk of bias is unclear

**Fig. 3:**
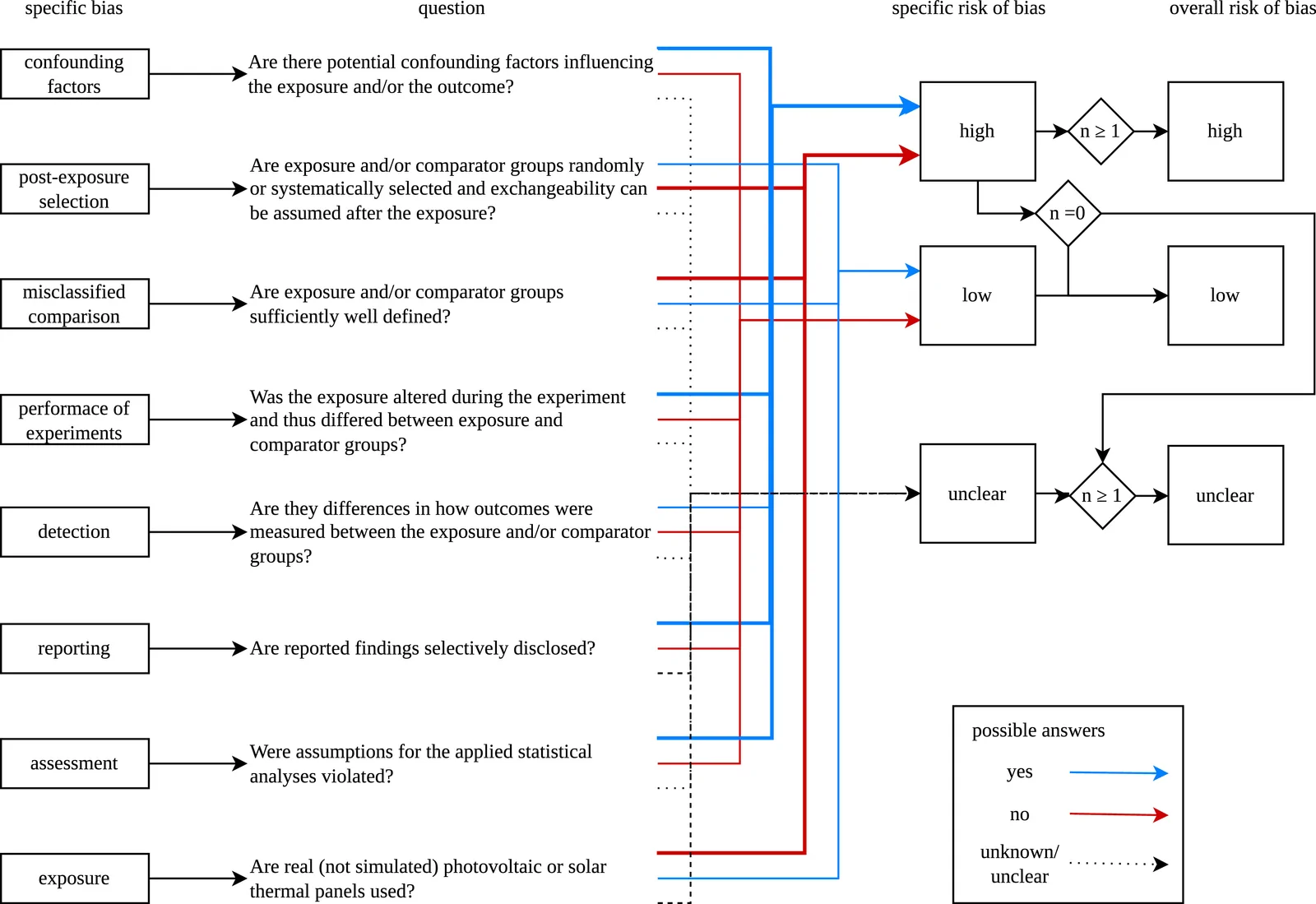
Flow diagram of the critical appraisal tool (CAT) by Lafitte et al. [2,26] for assessing the specific and overall risks of bias per study.

This is a rather strict overall risk of bias assessment compared with the possible CAT workflows described by Konno et al. [28].

h) **Data synthesis plan**

The results of the study selection, study coding, data extraction, and critical appraisal of studies were presented in a narrative synthesis of the related article with figures and tables . Quantitative analyses were included wherever possible, owing to the sample size and statistical requirements. The data corpus is illustrated using a chord diagram and discussed narratively.

## Protocol validation

The search string was validated using the pre-defined benchmark list and the published search string by Lafitte et al. [2], as described above.

## Limitations

We are aware that this review protocol cannot meet all the criteria of a systematic review by the acknowledged guidelines in ecology (e.g., CEE guidelines). The main reasons are time and resource constraints. The topic was chosen by the authors and not registered and advertised in a network. No advisory board including experienced systematic reviewers could be established because of limited time and financial resources. However, the review team including experienced scientists from different disciplines and institutions. In addition, two review team members were assigned the roles of “chairs” rather than conducting the review. We believe that a large review team and advisory board would not be essential for the narrow topic of this review.

Certainly, one major limitation is the lack of stakeholder engagement. Although, it would have been beneficial, potential stakeholders could not be involved at any level of this review process. Stakeholder involvement would have been especially beneficial when formulating the research question, identifying the search terms, and conducting critical appraisals. In addition, stakeholder engagement would likely increase the acceptance of the results of this review. However, stakeholders were involved at a minimum level without specific mapping: During the kickoff meeting of the research project facilitating this review, experts from administrative bodies and nature conservationists were present, and one topic of discussion was solar facilities and nature conservation, with a focus on organic (peat) soils in Germany. In addition, the review team attended a diverse range of online meetings and webinars on the topic of solar energy and nature conservation to gain a first overview of relevant questions, opinions, and potential lack of evidence. The broader topic of PV facilities and their impacts on nature conservation was discussed with practitioners, administrative bodies, and nature conservationists at conferences and online meetings. However, the focus of all these discussions was Germany.

In addition, the literature search and screening process focused on publications in English and German because of the linguistic abilities of the review team. No additional publications, for example, in French from Lafitte et al. [2], could be included in the narrative synthesis because the need to understand the content and interpretations was beyond the linguistic abilities of all reviewers. We believe that using AI tools for translation would be sufficient for screening; however, the coding, critical appraisal, and data extraction presume full understanding of the publication text, which is beyond the capabilities of current AI tools.

## Declaration of generative AI and AI-assisted technologies in the writing process

During the preparation of this work, the authors used Grammarly free version for basic grammar, spelling and punctuation checks to improve the manuscript. After using this tool, the authors reviewed and edited the content as needed and take full responsibility for the content of the publication.

## CRediT authorship contribution statement

**Wiltrut Koppensteiner:** Writing – original draft, Writing – review & editing, Conceptualization, Methodology, Resources, Data curation, Formal analysis, Validation, Visualization, Project administration. **Arndt Piayda:** Writing – review & editing, Conceptualization, Validation, Supervision. **Hanna Rae Martens:** Writing – review & editing, Validation. **Felix Zitzmann:** Writing – review & editing, Validation. **Kirsten Brandt:** Writing – review & editing, Validation. **Franziska Tanneberger:** Writing – review & editing, Supervision, Funding acquisition. **Bärbel Tiemeyer:** Writing – review & editing, Conceptualization, Supervision, Funding acquisition.

## Declaration of interests

The authors declare that they have no known competing financial interests or personal relationships that could have appeared to influence the work reported in this paper.

## Data Availability

Data will be made available on request.

## Appendix Supplementary materials

Supplementary material *and/or* additional information [OPTIONAL] MEX_SupplemataryMaterial_S1_spreadsheets.xlsx

MEX_SupplemataryMaterial_S2 - tables_figrues.docx
