# Supplementary material for: Systematic review protocol for evaluating the ecological effects of ground-mounted photovoltaic facilities on faunistic biodiversity in grasslands✰
Source: MethodsX. 2026 Oct 14;17:104209. doi: 10.1016/j.mex.2026.104209 (PMC13718679; doi:10.1016/j.mex.2026.104209)
Supplement: Supplementary file 1 [file mmc2.docx]

**Supplementary material:**

**Systematic review protocol for evaluating the ecological effects of ground-mounted photovoltaic facilities on faunistic biodiversity in grasslands**

Table S1: Identified potential search terms for the naïve search string (Grames et al., 2019), and terms that need to be excluded from search string. Boolean operators and Wildcards: * = any number of characters, incl. zero; “$” zero or one character only.

| **Term 1**  Population species | **OR** | **Term 2**  Population habitat | **AND** | **Term 3**  Exposure | **NOT** | **Term 4**  Exclude for population | **NOT** | **Term 5**  Exclude for exposure |
| --- | --- | --- | --- | --- | --- | --- | --- | --- |
| biodivers* |  | ecological |  | photovoltaic$ |  | plant spec* |  | roof* |
| OR species |  | OR conservation |  | OR PV |  | OR vegetation |  | OR house$ |
| OR wildlife |  |  |  | OR solar power |  | OR flora |  | OR building$ |
| OR fauna |  |  |  | OR solar power plant$ |  | OR desert |  |  |
| OR mammal$ |  |  |  | OR solar park$ |  |  |  |  |
| OR bird$ |  |  |  | OR solar farm$ |  |  |  |  |
| OR avian |  |  |  | OR solar facilit* |  |  |  |  |
| OR bat$ |  |  |  | OR solar panel |  |  |  |  |
| OR reptile$ |  |  |  |  |  |  |  |  |
| OR amphibian$ |  |  |  |  |  |  |  |  |
| OR insect$ |  |  |  |  |  |  |  |  |
| OR arachnid$ |  |  |  |  |  |  |  |  |
| OR arthropod$ |  |  |  |  |  |  |  |  |
| OR invertebrate$ |  |  |  |  |  |  |  |  |

Table S2: Search string for scoping of naïve literature search in the databases Web of Science Core Collection (filter = exact words). [conducted 15.01.2024 by WK]. Wildcards: * = any number of characters, incl. zero; “$” zero or one character only, “?” one character only and can be repeated. Text bold = additional search terms.

| **Scoping steps** | **Search string on topic (TS)** | **results WOSCC** | **adaptation comments** |
| --- | --- | --- | --- |
| Naïve search string basis | TS = ((photovoltaic$ OR PV OR “solar power” OR “solar power plant$” OR “solar park$” OR “solar farm$” OR “solar facilit*” OR “solar panel”)  AND  (biodivers* OR species OR wildlife OR fauna OR mammal$ OR bird$ OR avian OR bat$ OR reptile$ OR amphibian$ OR insect$ OR arachnid$ OR arthropod$ OR invertebrate$ OR “ecological” OR “conservation”)) | **8,840** | **Start of scoping** |
| Naïve search string scoping:  stepwise exclusion and adaption of terms | TS = ((photovoltaic$ OR PV OR “solar power” OR “solar power plant$” OR “solar park$” OR “solar farm$” OR “solar facilit*” OR “solar panel”)  AND  (**(((biodivers* OR species) NOT (plant spec* OR vegetation OR flora))** OR wildlife OR fauna OR mammal$ OR bird$ OR avian OR bat$ OR reptile$ OR amphibian$ OR insect$ OR arachnid$ OR arthropod$ OR invertebrate$ OR “ecological” OR “conservation”**)**)) | **7,363** | Exclude plant species: ((biodivers* OR species) NOT (plant spec* OR vegetation OR flora)) |
|  | TS = ((photovoltaic$ OR “solar power” OR “solar power plant$” OR “solar park$” OR “solar farm$” OR “solar facilit*” OR “solar panel”)  AND  ((((biodivers* OR species) NOT (plant spec* OR vegetation OR flora)) OR wildlife OR fauna OR mammal$ OR bird$ OR avian OR bat$ OR reptile$ OR amphibian$ OR insect$ OR arachnid$ OR arthropod$ OR invertebrate$ OR “ecological” OR “conservation”))) | **4,284** | Exclude ambiguous term “PV”, because not only photovoltaic but also plasma volume |
|  | TS = ((photovoltaic$ OR “solar power” OR “solar power plant$” OR “solar park$” OR “solar farm$” OR “solar facilit*” OR “solar panel”)  AND  ((((biodivers* OR species) NOT (plant spec* OR vegetation OR flora)) OR wildlife OR fauna OR mammal$ OR bird$ OR avian OR **chiroptera$** OR reptile$ OR amphibian$ OR insect$ OR arachnid$ OR arthropod$ OR invertebrate$ OR “ecological” OR “conservation”))) | **2,936** | Adapt ambiguous “bat” to exclude f.e. “BAT algorithm”, battery terms and chemical terms:  chiroptera$ instead |
|  | TS = ((photovoltaic$ OR “solar power” OR “solar power plant$” OR “solar park$” OR “solar farm$” OR “solar facilit*” OR “solar panel”)  AND  (((biodivers* NOT (plant spec* OR vegetation OR flora)) OR wildlife OR fauna OR mammal$ OR bird$ OR avian OR chiroptera$ OR reptile$ OR amphibian$ OR insect$ OR arachnid$ OR arthropod$ OR invertebrate$ OR “ecological” OR “conservation”))) | **1,371** | Exclude term “species”, because also ambiguous for technique terms |
|  | TS = ((photovoltaic$ OR “solar power” OR “solar power plant$” OR “solar park$” OR “solar farm$” OR “solar facilit*” OR “solar panel”)  AND  ((biodivers* OR wildlife OR fauna OR mammal$ OR bird$ OR avian OR chiroptera$ OR reptile$ OR amphibian$ OR insect$ OR arachnid$ OR arthropod$ OR invertebrate$ OR “ecological” OR “conservation”))) | **1,376** | Simplify search string, exclude NOT-cause for plants; no improvement, include NOT-cause again |
|  | TS = ((**(**photovoltaic$ OR “solar power” OR “solar power plant$” OR “solar park$” OR “solar farm$” OR “solar facilit*” OR “solar panel”**)** **NOT (roof* OR house$ OR building$)**)  AND  ((biodivers* OR wildlife OR fauna OR mammal$ OR bird$ OR avian OR chiroptera$ OR reptile$ OR amphibian$ OR insect$ OR arachnid$ OR arthropod$ OR invertebrate$ OR “ecological” OR “conservation”))) | **1,051** | include NOT-cause for plants again,  exclude photovoltaic on roofs etc.:  NOT (roof* OR house$ OR building$) |
|  | TS = (((photovoltaic$ OR “solar power” OR “solar power plant$” OR “solar park$” OR “solar farm$” OR “solar facilit*” OR “solar panel**”) NOT (roof* OR house$ OR building$ OR “solar cell$”)**)  AND  ((biodivers* OR wildlife OR fauna OR mammal$ OR bird$ OR avian OR chiroptera$ OR reptile$ OR amphibian$ OR insect$ OR arachnid$ OR arthropod$ OR invertebrate$ OR “ecological” OR “conservation”))) | **933** | Exclude terms “solar cells”, because very technical papers using this term:  NOT (roof* OR house$ OR building$ OR “solar cell$”) |
|  | TS = (((photovoltaic$ OR “solar power” OR “solar power plant$” OR “solar park$” OR “solar farm$” OR “solar facilit*” OR “solar panel”) **NOT (roof* OR house$ OR building$ OR “solar cell$” OR “satellite transmitter$”)**)  AND  ((biodivers* OR wildlife OR fauna OR mammal$ OR bird$ OR avian OR chiroptera$ OR reptile$ OR amphibian$ OR insect$ OR arachnid$ OR arthropod$ OR invertebrate$ OR “ecological” OR “conservation”))) | **931** | Exclude terms “satellite transmitter”, to try to exclude animal tracking with solar panels:  NOT (roof* OR house$ OR building$ OR “solar cell$” OR “satellite transmitter$”), but exclusion terms not sufficient to exclude animal tracking, delete term exclusion again |
|  | TS = (((photovoltaic$ OR “solar power” OR “solar power plant$” OR “solar park$” OR “solar farm$” OR “solar facilit*” OR “solar panel”) **NOT (roof* OR house$ OR building$ OR “solar cell$” OR “solar$powered” OR “light trap$”)**)  AND  ((biodivers* OR wildlife OR fauna OR mammal$ OR bird$ OR avian OR chiroptera$ OR reptile$ OR amphibian$ OR insect$ OR arachnid$ OR arthropod$ OR invertebrate$ OR “ecological” OR “conservation”))) | **930** | Exclude terms “solar powered” and “light trap”, to try to exclude animal tracking with solar panels:  NOT (roof* OR house$ OR building$ OR “solar cell$” OR “solar$powered” OR “light trap$”), but exclusion terms, delete term exclusion again |
|  | TS = (((photovoltaic$ OR “solar power” OR “solar power plant$” OR “solar park$” OR “solar farm$” OR “solar facilit*” OR “solar panel”) **NOT (roof* OR house$ OR building$ OR “solar cell$” OR “concentrated solar” OR “concentrating solar”)**)  AND  ((biodivers* OR wildlife OR fauna OR mammal$ OR bird$ OR avian OR **chiroptera$** OR reptile$ OR amphibian$ OR insect$ OR arachnid$ OR arthropod$ OR invertebrate$ OR “ecological” OR “conservation”))) | **878** | Exclude terms on “concentrated solar facilities”:  NOT (roof* OR house$ OR building$ OR “solar cell$” OR “concentrated solar” OR “concentrating solar”) |
|  | TS = (((photovoltaic$ OR “solar power” OR “solar power plant$” OR “solar park$” OR “solar farm$” OR “solar facilit*” OR “solar panel”) NOT (roof* OR house$ OR building$ OR “solar cell$” OR “concentrated solar” OR “concentrating solar”))  AND  ((biodivers* OR wildlife OR fauna OR mammal$ OR bird$ OR avian OR chiroptera$ OR reptile$ OR amphibian$ OR insect$ OR arachnid$ OR arthropod$ OR invertebrate$ OR “ecological” OR “conservation”)) **NOT desert)** | **844** | Exclude desert habitats:  NOT desert |
| Naïve search string scoping:  strings for the comparison with Lafitte et al. (2023) search string; | TS = (((photovoltaic$ OR “solar power” OR “solar power plant$” OR “solar park$” OR “solar farm$” OR “solar facilit*” OR “solar panel”) NOT (roof* OR house$ OR building$ OR “solar cell$” OR “concentrated solar” OR “concentrating solar”))  AND  ((biodivers* OR wildlife OR fauna OR mammal$ OR bird$ OR avian OR chiroptera$ OR reptile$ OR amphibian$ OR insect$ OR arachnid$ OR arthropod$ OR invertebrate$ OR **“conservation”**))) | **565** | Exclude term “ecological”, simplify search string by deleting NOT-causes:  NOT desert |
|  | TS = ((photovoltaic$ OR “solar power” OR “solar power plant$” OR “solar park$” OR “solar farm$” OR “solar facilit*” OR “solar panel”)  AND  (biodivers* OR wildlife OR fauna OR mammal$ OR bird$ OR avian OR chiroptera$ OR reptile$ OR amphibian$ OR insect$ OR arachnid$ OR arthropod$ OR invertebrate$ OR **“conservation”**)) | **938** | Simplify search string by deleting NOT-causes:  NOT (roof* OR house$ OR building$ OR “solar cell$” OR “concentrated solar” OR “concentrating solar”) |
|  | TS = (((photovoltaic$ OR “solar power” OR “solar power plant$” OR “solar park$” OR “solar farm$” OR “solar facilit*” OR “solar panel”) **NOT (roof* OR house$ OR building$ OR “solar cell$” OR “concentrated solar” OR “concentrating solar”)**)  AND  ((biodivers* OR wildlife OR fauna OR mammal$ OR bird$ OR avian OR chiroptera$ OR reptile$ OR amphibian$ OR insect$ OR arachnid$ OR arthropod$ OR invertebrate$ OR **“ecological”**))) | **553** | include term “ecological” and exclude “conservation”,  add NOT-causes again:  NOT (roof* OR house$ OR building$ OR “solar cell$” OR “concentrated solar” OR “concentrating solar”) |
|  | TS = ((photovoltaic$ OR “solar power” OR “solar power plant$” OR “solar park$” OR “solar farm$” OR “solar facilit*” OR “solar panel”)  AND  (biodivers* OR wildlife OR fauna OR mammal$ OR bird$ OR avian OR chiroptera$ OR reptile$ OR amphibian$ OR insect$ OR arachnid$ OR arthropod$ OR invertebrate$ OR **“ecological”**)) | **772** | Simplify search string by deleting NOT-causes:  NOT (roof* OR house$ OR building$ OR “solar cell$” OR “concentrated solar” OR “concentrating solar”) |
|  | TS = (((photovoltaic$ OR “solar power” OR “solar power plant$” OR “solar park$” OR “solar farm$” OR “solar facilit*” OR “solar panel”) **NOT (roof* OR house$ OR building$ OR “solar cell$” OR “concentrated solar” OR “concentrating solar”)**)  AND  ((biodivers* OR wildlife OR fauna OR mammal$ OR bird$ OR avian OR chiroptera$ OR reptile$ OR amphibian$ OR insect$ OR arachnid$ OR arthropod$ OR invertebrate$))) | **219** | Exclude both terms “ecological”and “conservation”,  add NOT-causes again:  NOT (roof* OR house$ OR building$ OR “solar cell$” OR “concentrated solar” OR “concentrating solar”) |
|  | TS = ((photovoltaic$ OR “solar power” OR “solar power plant$” OR “solar park$” OR “solar farm$” OR “solar facilit*” OR “solar panel")  AND  (biodivers* OR wildlife OR fauna OR mammal$ OR bird$ OR avian OR chiroptera$ OR reptile$ OR amphibian$ OR insect$ OR arachnid$ OR arthropod$ OR invertebrate$)) | **307** | Simplify search string by deleting NOT-causes:  NOT (roof* OR house$ OR building$ OR “solar cell$” OR “concentrated solar” OR “concentrating solar”) |
| search string of Lafitte et al. 2023; | TS = ((photovoltaic$ OR “solar panel$” OR “solar array$” OR “solar development$” OR “solar power” OR “solar park$” OR “solar installation$” OR “solar facilit*” OR “solar plant$” OR “utility-scale solar energ*” OR “utility scale solar energ*” OR biosolar OR “float* solar” OR floatovoltaic$) AND (biodiversity OR ecolog* OR ecosystem$ OR wildlife OR “natural habitat$” OR species OR flora OR vegetation$ OR animal$ OR fauna OR vertebrate$ OR mammal$ OR bird$ OR reptile$ OR amphibian$ OR invertebrate$ OR arthropod$ OR insect$ OR arachnid$ OR crustacean$ OR mollus* OR microbi* OR bacteri* OR microorganism$ OR fung*)) | **3,670** | Irrelevant publications on about tracking systems, concentrated solar power, green roofs, PV on roofs included |

Table S3 selection of search terms based on PEO. The concept group “Outcome” was not included in the systematic search string. Manually added search terms are marked with (+).

| groups | population | exposure | outcome |
| --- | --- | --- | --- |
| terms | aquatic animals | agrivoltaic systems | animal behaviour |
|  | aquatic insects | agrivoltaics | case studies |
|  | aquatic organisms | floating photovoltaic | ecological impact |
|  | biodiversity | floating photovoltaic systems | ecological impacts |
|  | conservation | floating solar | ecological restoration |
|  | ecological | ground-mounted photovoltaic | effects |
|  | ecology | photovoltaic | environmental assessment |
|  | ecosystem | photovoltaic arrays | environmental degradation |
|  | ecosystems | photovoltaic installations | environmental impact |
|  | environment | photovoltaic panels | environmental impacts |
|  | nature conservation | photovoltaic power plant | habitat loss |
|  | species | photovoltaic power plants | habitat suitability |
|  | wild animals | photovoltaic solar | population density |
|  | wildlife | photovoltaic solar energy | species abundance |
|  | fauna (+) | photovoltaic solar farms | species composition |
|  | bird$ (+) | photovoltaic solar panel | species diversity |
|  | avian (+) | photovoltaics | species richness |
|  | amphibian$ (+) | solar energy facilities | survival |
|  | chiroptera (+) | solar facilities |  |
|  | reptile$ (+) | solar farm |  |
|  | insect$ (+) | solar farms |  |
|  |  | solar panel |  |
|  |  | solar panels |  |
|  |  | solar parks |  |
|  |  | solar photovoltaic |  |
|  |  | solar photovoltaics |  |
|  |  | solar plants |  |
|  |  | utility scale solar |  |
|  |  | utility-scale solar |  |
|  |  | utility-scale solar energy |  |
|  |  | Solar facility (+) |  |
|  |  | Solar park (+) |  |
|  |  | Solar power plant$ (+) |  |
|  |  | Solar power park$ (+) |  |

Figure S1: Document feature matrix of unique keywords overview generated by applying the pruned naïve search results in the R-package litsearchr (Grames et al., 2019) using built (min_studies = 2, min_occ = 2). All keywords are in one cluster.

Figure S2: Strengths of the terms by rank. Red line = cumulative cut off 70%, orange line = change point cutoffs (85, 210, 341, 701); min. rank = weakest link between terms, max. rank = strongest link between terms.

Table S4: Adaptation steps of the systematic built search string from litsearchr (Grames et al., 2019). In red are terms that are adapted in the next step. Bold are the steps to be tested for comprehensiveness. Red symbols/ words are removed in next step.

| **adaptation step** | **Search string** | **Hits in WOSCC** |
| --- | --- | --- |
| **Naïve search string (scoping result)** | TS = ((photovoltaic$ OR “solar power” OR “solar power plant$” OR “solar park$” OR “solar farm$” OR “solar facilit*” OR “solar panel”) AND (biodivers* OR wildlife OR fauna OR mammal$ OR bird$ OR avian OR chiroptera$ OR reptile$ OR amphibian$ OR insect$ OR arachnid$ OR arthropod$ OR invertebrate$ OR “ecological”)) | WOSCC: 772 |
| **Naïve search string with additional term** | TS = (biodivers* AND (photovoltaic$ OR “solar power” OR “solar power plant$” OR “solar park$” OR “solar farm$” OR “solar facilit*” OR “solar panel”) AND (biodivers* OR wildlife OR fauna OR mammal$ OR bird$ OR avian OR chiroptera$ OR reptile$ OR amphibian$ OR insect$ OR arachnid$ OR arthropod$ OR invertebrate$ OR “ecological”)) | WOSCC: 107 |
| **Original *litsearchr* search string** | TS = (("solar* facil*" OR "solar* park*" OR "solar* power* park*" OR "solar* plant*" OR "solar* power* plant*" OR "agrivolta* system*" OR agrivolta* OR "float* solar*" OR photovolta* OR "solar* energi* facil*" OR "solar* farm*" OR "solar* panel*" OR "util* scale* solar*" OR "utility-scal* solar*") AND (fauna* OR bird* OR avian* OR amphibian* OR chiroptera* OR reptil* OR insect* OR "aquat* anim*" OR "aquat* organ*" OR biodivers* OR conserv* OR ecolog* OR ecosystem* OR environ* OR speci* OR "wild* anim*" OR wildlif*)) | WOSCC: 28,173 |
| **Revision of stemmed terms for confounding** | TS = (("solar facil*" OR "solar park*" OR "solar power park*" OR "solar plant*" OR "solar power plant*" OR "agrivolta* system*" OR agrivolta* OR "float* solar" OR photovolta* OR "solar energy facil*" OR "solar farm*" OR "solar panel*" OR "utility scale* solar" OR "utility-scale* solar") AND (fauna OR bird* OR avian OR amphibian* OR chiroptera OR reptil* OR insect* OR "aquat* anim*" OR biodivers* OR conserv* OR ecolog* OR ecosystem* OR environ* OR speci* OR "wild anim*" OR wildlife)) | WOSCC: 28,176 |
| **Adapted *litsearchr* search string** | TS = (("solar facil*" OR "solar park*" OR "solar power park*" OR "solar plant*" OR "solar power plant*" OR "agrivolta* system*" OR agrivolta* OR photovolta* OR "solar energy facil*" OR "solar farm*" OR "solar panel*" OR "utility scale* solar" OR "utility-scale* solar") AND (fauna OR bird* OR avian OR amphibian* OR chiroptera OR reptil* OR insect* OR "aquat* anim*" OR biodivers* OR ecolog* OR "wild anim*" OR wildlife) NOT “life cycle”) | WOSCC: 800 |
| **Adapted *litsearchr* search string with additional term** | TS = (**biodivers* AND** ("solar* facil*" OR "solar* park*" OR "solar* power* park*" OR "solar* plant*" OR "solar* power* plant*" OR "agrivolta* system*" OR agrivolta* OR "float* solar*" OR photovolta* OR "solar* energi* facil*" OR "solar* farm*" OR "solar* panel*" OR "util* scale* solar*" OR "utility-scal* solar*") AND (fauna* OR bird* OR avian* OR amphibian* OR chiroptera* OR reptil* OR insect* OR "aquat* anim*" OR "aquat* organ*" OR biodivers* OR conserv* OR ecolog* OR ecosystem* OR environ* OR speci* OR "wild* anim*" OR wildlif*)) | WOSCC: 108 |

Table S5: List of applied search strings in multiple databases using the adapted systematic search string. Database specific adaptations were required.

| Search string | Database or further sources | Results | Date | Comments |
| --- | --- | --- | --- | --- |
| TITLE-ABS-KEY (("solar facil*" OR "solar park*" OR "solar power park*" OR "solar plant*" OR "solar power plant*" OR "agrivolta* system*" OR agrivolta* OR photovolta* OR "solar energy facil*" OR "solar farm*" OR "solar panel*" OR "utility scale* solar" OR "utility-scale* solar" AND NOT “life cycle”) AND (fauna OR bird* OR avian OR amphibian* OR chiroptera OR reptil* OR insect* OR "aquat* anim*" OR biodivers* OR ecolog* OR "wild anim*" OR wildlife)) | SCOPUS | 2368 | 2024-03-13 | academic |
| TS = (("solar facil*" OR "solar park*" OR "solar power park*" OR "solar plant*" OR "solar power plant*" OR "agrivolta* system*" OR agrivolta* OR photovolta* OR "solar energy facil*" OR "solar farm*" OR "solar panel*" OR "utility scale* solar" OR "utility-scale* solar") AND (fauna OR bird* OR avian OR amphibian* OR chiroptera OR reptil* OR insect* OR "aquat* anim*" OR biodivers* OR ecolog* OR "wild anim*" OR wildlife) NOT “life cycle”) | ProQuest | 100 | 2024-03-13 | theses |
| allintitle: (wildlife\|"wild animals") (photovoltaic\|"solar farm"\|"solar park"\|"solar facility") -"life cycle" allintitle: biodiversity (photovoltaic\|"solar farm"\|"solar park"\|"solar facility") -"life cycle" allintitle: fauna (photovoltaic\|"solar farm"\|"solar park"\|"solar facility") -"life cycle" allintitle: bird (photovoltaic\|"solar farm"\|"solar park"\|"solar facility") -"life cycle" | Google Scholar | 71 | 2024-03-13 | grey |
| Biodiversität ("Freiflächen-PV" \| "Photovoltaik Freiflächenanlagen") Plus: Fauna ("Freiflächen-PV" \| "Photovoltaik Freiflächenanlagen") | Google Scholar | 234 | 2024-03-13 | grey de |
| Biodiversität + Freiflächen-PV Plus: Biodiversität + Solarpark Biodiversität + Photovoltaik + Freiflächen | BfN e-repositorium | 10 | 2024-03-14 | grey |
| none | GMC Publications | 2 | 2024-03-13 | grey |
| none | KNE | 12 | 2024-03-13 | grey |
| TS = (("solar facil*" OR "solar park*" OR "solar power park*" OR "solar plant*" OR "solar power plant*" OR "agrivolta* system*" OR agrivolta* OR photovolta* OR "solar energy facil*" OR "solar farm*" OR "solar panel*" OR "utility scale* solar" OR "utility-scale* solar") AND (fauna OR bird* OR avian OR amphibian* OR chiroptera OR reptil* OR insect* OR "aquat* anim*" OR biodivers* OR ecolog* OR "wild anim*" OR wildlife) NOT “life cycle”) | Web of Science Core Collection | 830 | 2024-03-13 | academic |
| TS = (("solar facil*" OR "solar park*" OR "solar power park*" OR "solar plant*" OR "solar power plant*" OR "agrivolta* system*" OR agrivolta* OR photovolta* OR "solar energy facil*" OR "solar farm*" OR "solar panel*" OR "utility scale* solar" OR "utility-scale* solar") AND (fauna OR bird* OR avian OR amphibian* OR chiroptera OR reptil* OR insect* OR "aquat* anim*" OR biodivers* OR ecolog* OR "wild anim*" OR wildlife) NOT “life cycle”) | CAB Abstract | 380 | 2024-03-13 | academic |
| TS = (("solar facil*" OR "solar park*" OR "solar power park*" OR "solar plant*" OR "solar power plant*" OR "agrivolta* system*" OR agrivolta* OR photovolta* OR "solar energy facil*" OR "solar farm*" OR "solar panel*" OR "utility scale* solar" OR "utility-scale* solar") AND (fauna OR bird* OR avian OR amphibian* OR chiroptera OR reptil* OR insect* OR "aquat* anim*" OR biodivers* OR ecolog* OR "wild anim*" OR wildlife) NOT “life cycle”) | Biological Abstract | 570 | 2024-03-13 | academic |
| TS = (("solar facil*" OR "solar park*" OR "solar power park*" OR "solar plant*" OR "solar power plant*" OR "agrivolta* system*" OR agrivolta* OR photovolta* OR "solar energy facil*" OR "solar farm*" OR "solar panel*" OR "utility scale* solar" OR "utility-scale* solar") AND (fauna OR bird* OR avian OR amphibian* OR chiroptera OR reptil* OR insect* OR "aquat* anim*" OR biodivers* OR ecolog* OR "wild anim*" OR wildlife) NOT “life cycle”) | BIOSIS Citation Index | 524 | 2024-03-13 | academic |

**References**

Grames EM, Stillman AN, Tingley MW, Elphick CS. 2019. An automated approach to identifying search terms for systematic reviews using keyword co-occurrence networks. Methods in Ecology and Evolution **10**:1645–1654.
